# Optimizing nitrogen fertilizer efficacy under narrow irrigation limit range: a synergistic approach to okra nutrient management

**DOI:** 10.1186/s12870-026-08194-6

**Published:** 2026-01-30

**Authors:** Shenghui Xu, Yunxiang Huang, Huaiyu Long, Li Niu, Hongjie Ji

**Affiliations:** 1 State Key Laboratory of Efffcient Utilization of Arid and Semi-Arid Arable Land in Northern China, Institute of Agricultural Resources and Regional Planning, Chinese Academy of Agricultural Sciences, 12 South Road, Zhongguancun, Haidian District, Beijing, 100081 China; 2 State Key Laboratory of Crop Genetics and Germplasm Enhancement, Nanjing Agricultural University, Nanjing, 210095 China; 3 College of Resources, Hunan Agricultural University, Changsha, 410128 China; 4 Beijing Changping Agricultural Technology Extension Station, Beijing, 102200 China

**Keywords:** Okra, Irrigation limit ranges, Nitrogen application, Fruit quality, Soil enzyme activities

## Abstract

Optimizing water and nitrogen (N) management is critical for enhancing crop productivity under water scarcity. This study aimed to determine if a high irrigation upper limit can compensate for a low lower limit and whether optimal N application can mitigate the impacts of a wide irrigation range in okra. A pot experiment was conducted with three irrigation ranges (defined by the lower and upper limits of soil field capacity, FC) and two N rates: W1 (45–55% FC), W2 (35–65% FC), W3 (25–75% FC) and N1 (110 kg ha⁻1), N2 (220 kg ha⁻1). Results demonstrated that a high upper limit could not compensate for the adverse effects of a low lower limit. The narrow irrigation range (W1) consistently outperformed wider ranges, increasing yield by up to 42.3% compared to the wide irrigation range (W3). Moderate N application (N1) effectively alleviated the impacts associated with W3, boosting its yield by 24.9%, whereas excessive N (N2) was often detrimental. The optimal W1N1 combination synergistically enhanced system performance, achieving the highest yield (56.7% greater than the poorest N2W3 treatment), improving fruit quality (e.g., 11.5% higher soluble sugar), and increasing soil urease activity by 25.9%. Conversely, N2 led to soil acidification and nutrient imbalance. These results demonstrate that coupling a narrow irrigation limit range with moderate N is an optimal strategy for enhancing okra productivity and soil health, providing a viable guide for sustainable cultivation.

## Introduction

Soil moisture is a fundamental driver of crop growth in agricultural ecosystems. It regulates key plant physiological processes and governs the movement of nutrients within the soil, ultimately determining crop development and yield [6, 55, 61]. Appropriate irrigation management has long been recognized as critical for ensuring stable and high crop yields [27, 35]. However, increasing climate variability has resulted in more frequent drought events and irregular precipitation patterns, leading to substantial fluctuations in soil water availability [15, 33, 36]. These changes pose serious challenges to sustainable agricultural water use, particularly in water-scarce regions. Therefore, understanding crop responses to varying soil moisture levels has become essential for optimizing irrigation strategies.

Okra (*Abelmoschus esculentus* L. Moench), a water-sensitive vegetable crop, is highly susceptible to soil moisture deficits [4]. Water stress significantly impairs okra growth and reduces fruit yield [7, 52]. Rewatering after a drought can facilitate a partial recovery in plant growth, a phenomenon known as the water-deficit compensation effect [11, 29]. For instance, crops like cotton and maize have demonstrated recovery of photosynthetic capacity upon rewatering [19, 37]. In irrigation management, this concept can be explored by defining an “irrigation range” between a lower limit (the soil moisture level that triggers irrigation) and an upper limit (the target moisture level after re-watering), both expressed as a percentage of field capacity (FC). Despite existing evidence on compensation effects, it remains unclear whether a high upper limit can effectively compensate for the adverse impacts of a low lower limit on okra. Addressing this question is critical for optimizing irrigation strategies under limited water availability.

Nitrogen (N) fertilization also plays a pivotal role in modulating plant responses to water stress. Adequate N application can enhance drought resilience by promoting root growth and maintaining photosynthetic activity [3, 17, 56]. Conversely, inappropriate N levels can exacerbate stress [2, 50]. Therefore, a significant research gap exists regarding whether appropriate N application can alleviate the adverse effects of expanded irrigation limits on okra.

Soil enzymatic activity serves as a sensitive indicator of microbial functionality and nutrient cycling. Enzymes such as urease, sucrase, alkaline phosphatase, and catalase respond rapidly to changes in soil moisture and N availability [34, 41, 59]. However, the combined effects of irrigation range and N levels on soil enzyme dynamics in okra-growing systems are not well-quantified.

The novelty of this research lies in the integrated investigation of the interaction between a defined narrow irrigation range and N rates, focusing on its synergistic effects on okra productivity, fruit quality, and soil enzyme activities. Therefore, this study aims to (i) determine whether a higher irrigation upper limit can compensate for the adverse effects of a low lower limit, and (ii) evaluate whether appropriate N fertilizer application can mitigate the negative impacts of a wide irrigation range. The findings will provide insights for optimizing water and N management to enhance resource use efficiency in okra production.

## Materials and methods

### Experimental site, soil preparation, and basic properties

This pot experiment was conducted from May to September 2021 at the research station of the College of Resources, Hunan Agricultural University (28°11′ N, 113°4′ E). A rain-proof canopy was installed to exclude natural precipitation and ensure precise control of soil moisture regimes.

The soil, classified as fluvo-aquic, was collected from the surface layer (0–20 cm) of the university’s cultivation garden. After collection, the soil was air-dried, crushed, and homogenized. For the pot experiment, the soil was sieved through a 0.5 cm mesh. Each culture pot, constructed from rubber and measuring 30 cm (top diameter) × 22 cm (bottom diameter) × 24 cm (height), was filled with 12 kg of the prepared soil, which was packed to achieve a bulk density equivalent to the measured field value of 1.1 g cm^–3^.

A separate 500 g subsample of the air-dried soil was sieved (through 2 mm and 0.149 mm meshes) for analysis of fundamental physicochemical properties. The soil exhibited a loamy clay texture, with a bulk density of 1.1 g cm^–3^ and a field capacity of 27.6% (v/v). The particle size distribution and chemical properties of the soil are presented in Table 1.

**Table 1:** Soil particle distribution and physicochemical properties

Particulars	Values
Clay (%)	31.5
Silt (%)	25.2
Sand (%)	43.2
Textural class	Loamy clay
Physical Properties	
Bulk density (g cm^–3^)	1.1
Chemical properties	
Soil pH (1:5 soil: water ratio)	5.53
Total nitrogen (g kg^–1^)	1.28
Total phosphorus (g kg^–1^)	0.84
Alkali hydrolyzed nitrogen (mg kg^–1^)	164
Available phosphorus (mg kg^–1^)	10.3
Available potassium (mg kg^–1^)	200
Organic matter (g kg^–1^)	19.6

### Experimental design and treatments

**Fig. 1:**
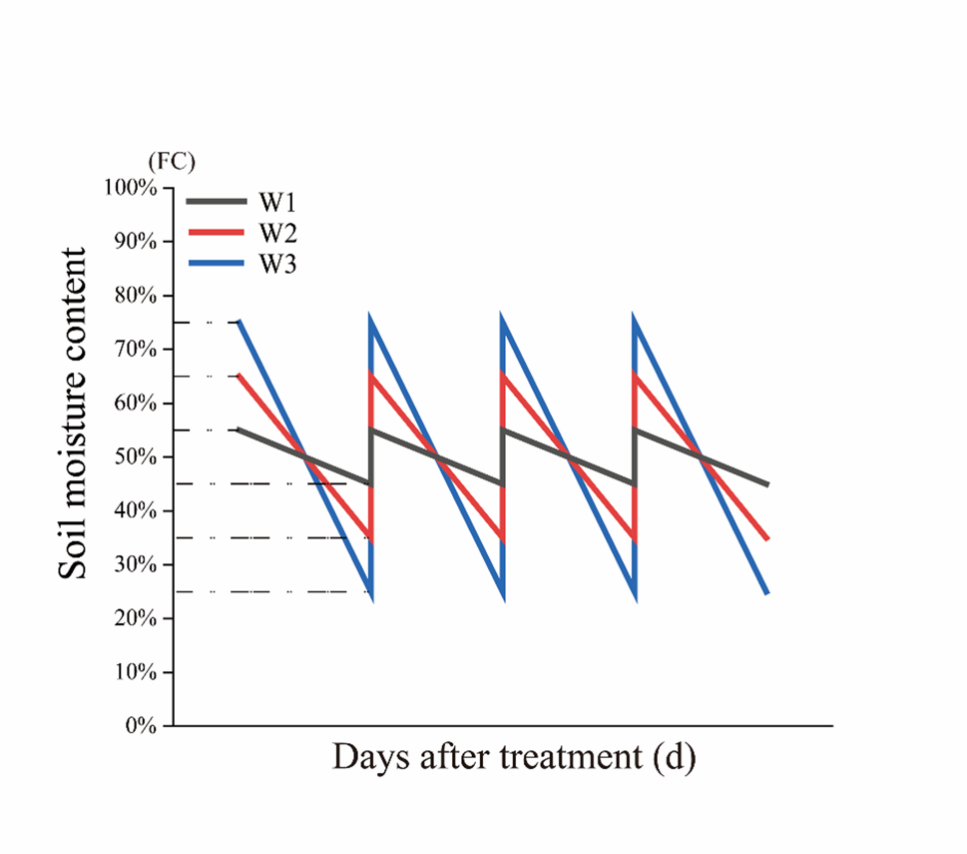
Schematic diagram of upper and lower limits of irrigation

The study employed a two-factor completely randomized design. The factors were irrigation limit (W) and nitrogen (N) application rate. Three irrigation ranges were established (Fig. 1) [58], centered around a median value considered optimal for okra growth [38]: W1 (narrow range) at 45–55% of field capacity (FC), W2 (moderate range) at 35–65% FC, and W3 (wide range) at 25–75% FC. For N levels, two application rates were used: N1 (moderate) at 110 kg N ha⁻¹ [10] and N2 (excessive) at 220 kg N ha⁻^1^. The factorial combination of three moisture regimes and two nitrogen levels resulted in six distinct treatments, each replicated four times, requiring a total of 24 pots arranged randomly.

### Crop establishment and management

Okra seeds (‘Wujiao’ variety, sourced from Weifang, Shandong Province) were sown in a seedling nursery on May 18, 2021. On June 8, 2021, uniform seedlings at the one-leaf-one-heart stage were transplanted into the pots. Initial soil moisture was maintained at 60% FC to promote seedling establishment. The designated differential moisture treatments (W1, W2, W3) commenced when the plants reached the three-leaf-one-heart stage. All necessary permissions and compliance with institutional and national guidelines were obtained.

### Fertilizer application

The following fertilizers were used: ammonium sulfate (N, 21%) as the nitrogen source, potassium phosphate (P₂O₅, 52%; K₂O, 35%) as the phosphorus source, and a combination of potassium sulfate (K₂O, 52%) and potassium phosphate as potassium sources.

The fertilization strategy was as follows: the entire amount of phosphorus fertilizer and 60% of the total nitrogen and potassium fertilizers were applied as a basal dose. Specifically, for the nitrogen treatments, ammonium sulfate was applied at rates of 5.7 g pot⁻¹ (N1) and 11.4 g pot⁻¹ (N2) as basal fertilizer. These were dissolved in water and thoroughly mixed into the soil before potting. The remaining 40% of the N and K fertilizers was applied in two equal splits (20% each) during the seedling and flowering stages. For N, this corresponded to topdressing applications of 1.14 g pot⁻¹ (N1) and 2.28 g pot⁻¹ (N2) of ammonium sulfate at each stage (seedling and flowering).

### Irrigation management

Soil moisture was monitored daily using a portable soil moisture meter (SU-LB, Beijing Meng Chuang Wei Ye Technology Co., Ltd. China). When the moisture content dropped to the lower limit of a given treatment, deionized water was added manually to restore it to the upper limit [58]. The experiment concluded on September 19, 2021, with the collection of rhizosphere soil samples for subsequent analysis.

### Sampling and measurements

#### Determination of agronomic traits, dry matter content, and yield

Growth indicators of okra were measured at the seedling stage (June 23rd) and flowering stage (July 28th). Plant height was determined as the distance from the base of the stem to the highest growth point of the plant, stem diameter was measured at the first internode above the cotyledon node, and the number of expanded leaves was determined by direct counting. After harvest, samples were separated by plant parts (including stems, leaves, and roots), heat-inactivated at 105 °C for 30 min, and dried to a constant weight at 80 °C to determine the dry matter content. The harvest yield of each plant was recorded, and the average value of four replicates was calculated.

#### Calculation of nitrogen partial factor productivity (NPFP)

The NPFP was calculated as follows [31]:

$$NPFP\;\left(\mathrm{kg}\;\mathrm{kg}^{-1}\right)=\;\mathrm Y/\mathrm N$$

where *Y* represents the yield in the nitrogen-applied area (kg ha⁻¹) and *N* denotes the amount of nitrogen applied (kg ha⁻¹).

#### Determination of okra quality indicators

The soluble sugar (SS) content was determined using the Anthrone colorimetric method [5]; the soluble protein (SP) content was measured using the Coomassie Brilliant Blue colorimetric assay [5];total soluble solids (TSS) were determined with an Atago Palette PR-32 (Atago Co., Ltd., Tokyo, Japan) digital refractometer [44]; the titratable acid soluble solids (TA) content was determined using the acid-base titration method (sodium hydroxide neutralization method) [44]; the flavonoid (F) content was measured using the sodium nitrite-aluminum nitrate-sodium hydroxide colorimetric method [5].

#### Determination of nutrient content in rhizosphere soil

Rhizosphere soil samples for each plant were collected following the shaking-off method [41], homogenized, bagged, and then brought back to the laboratory for analysis.

The contents of total nitrogen (TN), total phosphorus (TP), and ammonium (NH_4_^+^-N) nitrogen were determined by continuous flow analysis (AutoAnalyzer 3, sensitivity 0.001 AUFS; Bran & Luebbe, GmbH, Norderstedt, Germany) [23]. The total potassium (TK) and available potassium (AK) were determined by flame photometry method [46]. The soil organic matter (OM) content was measured by the potassium dichromate external heating method [46]. The available phosphorus (AP) was determined by molybdenum antimony colorimetry [30].

Soil pH was measured in a 1:2.5 (*w/v*) soil–deionized-water suspension, whereas EC was determined at a 1:5 ratio. After hand-shaking for 30 min, pH was read with a portable PHBJ-260 pH meter (0.01 pH resolution; Shanghai Precision & Scientific Instrument, Shanghai, China) and EC with an electrical conductivity meter (Leici DDS-307 A, Shanghai, China), following [46].

#### Determination of soil enzyme activity

Soil samples were collected one week after fertilization at the seedling and flowering stages. The samples were taken from a depth of 0–20 cm, 10 cm away from the base of the okra root stem, using a five-point sampling method and sieved through a 2 mm mesh. The activity of soil urease was determined using the sodium phenolate-hypochlorite indigo colorimetric method; the activity of soil sucrase was determined using the 3,5-dinitrosalicylic acid colorimetric method; the activity of soil alkaline phosphatase was determined using the disodium phenyl phosphate colorimetric method; and the activity of soil catalase was determined using the potassium permanganate titration method [21, 39, 49].

### Statistical analysis

Statistical analyses were performed using SPSS 22.0 (SPSS, Chicago, IL, USA). Prior to parametric analysis, all data were examined for normality with the Shapiro-Wilk test and for homogeneity of variances with Levene’s test. A two-way analysis of variance (ANOVA) was applied, followed by Duncan’s multiple-range test for multiple comparisons, with statistical significance set at *P* < 0.05. Figures were generated using Origin Pro 2021 (OriginLab Corporation, Northampton, MA, USA).

To investigate the relationships among agronomic traits, fruit quality, soil nutrients, soil enzymes, yield, and nitrogen partial factor productivity (NPFP), a partial least squares path model (PLS-PM) was constructed using the “plspm” package in R (version 4.1.1;Sanchez et al., [40]. The model’s quality was assessed through the goodness-of-fit index (GOF), where a value greater than 0.7 signifies an acceptable predictive performance of the model [8].

## Results

### Soil moisture dynamics and treatment verification

**Fig. 2:**
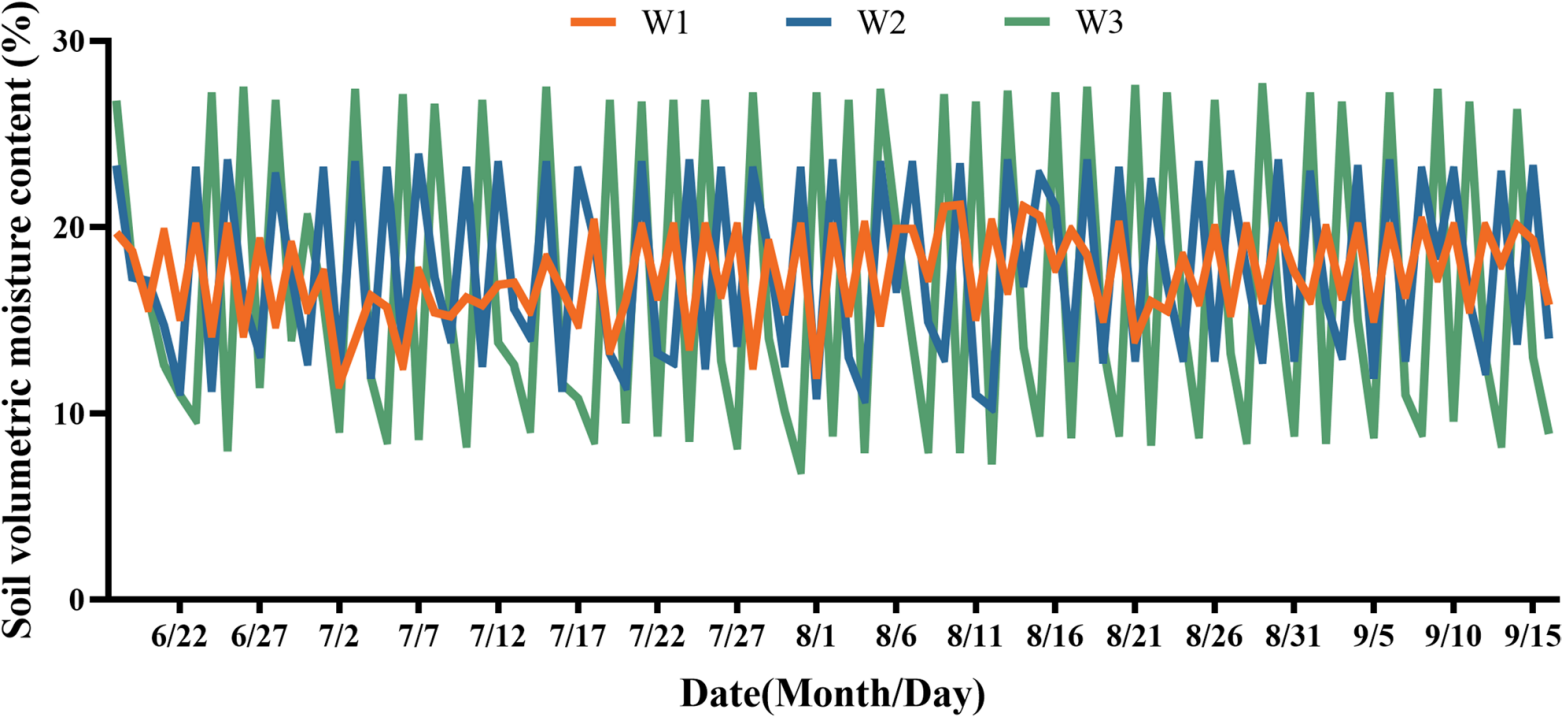
Temporal dynamics of actual soil volumetric water content under different irrigation regimes. Data points represent daily mean values for the W1 (narrow range), W2 (moderate range), and W3 (wide range) treatments over the experimental period

The effectiveness of the imposed irrigation regimes was verified daily with a portable soil moisture meter. As presented in Fig. 2, the actual volumetric water content successfully tracked the predefined fluctuation ranges throughout the experimental period. Specifically, the W1 treatment kept soil moisture between 12.4% and 15.2%, effectively realizing the designed narrow range of 45–55% of field capacity (FC). The W2 treatment exhibited fluctuations from 9.7% to 18.0%, corresponding to the moderate target range (35–65% FC). For the W3 treatment, soil moisture varied widely from 7.0% to 20.8%, achieving the intended wide fluctuation spectrum (25–75% FC). These data demonstrate that the combination of daily monitoring and manual irrigation was a reliable method for precisely implementing the distinct soil moisture fluctuation regimes outlined in the experimental design (Fig. 1).

### Agronomic traits and dry matter content

According to the ANOVA, irrigation limit (W), nitrogen (N) rate, and their interaction (W×N) had significant (*p* < 0.05) or highly significant (*p* < 0.01) effects on all measured agronomic traits and dry matter content at both seedling and flowering stages (Table 2).

**Table 2:** Effects of W and N application treatments on dry matter content of okra (g)

Treatments		Plant height (cm)		Stem diameter (mm)		Number of expanded leaves		Dry matter content (g)		
		s	f	s	f	s	f	Leaf	Stem	Root
N1	W1	13.3 ± 0.5a	43.5 ± 1.1a	5.03 ± 0.3a	14.1 ± 0.3a	6.00 ± 0.8a	15.0 ± 0.8a	83.0 ± 3.2a	32.2 ± 2.2a	11.5 ± 0.3a
	W2	12.7 ± 0.5ab	40.7 ± 0.9bc	4.50 ± 0.2b	14.1 ± 0.7a	5.00 ± 0.0ab	13.3 ± 0.9abc	72.4 ± 2.0b	29.9 ± 1.0ab	10.5 ± 0.8b
	W3	12.2 ± 0.2bc	39.8 ± 0.6c	3.93 ± 0.2c	12.4 ± 0.7b	4.67 ± 0.5b	12.0 ± 2.2bc	63.9 ± 4.4bc	24.3 ± 2.3c	7.5 ± 0.6c
N2	W1	12.7 ± 0.5ab	42.3 ± 0.5ab	4.03 ± 0.1c	13.2 ± 0.6ab	5.33 ± 0.5ab	14.0 ± 0.8ab	72.6 ± 2.2b	27.5 ± 0.1bc	7.5 ± 0.2c
	W2	12.3 ± 0.5bc	40.3 ± 0.5c	3.87 ± 0.2c	13.1 ± 0.6ab	5.00 ± 0.0ab	13.0 ± 0.8abc	63.1 ± 3.4c	24.5 ± 0.9c	7.2 ± 0.2c
	W3	11.7 ± 0.5c	39.2 ± 1.2c	3.63 ± 0.4c	11.0 ± 0.4c	4.67 ± 0.5b	10.7 ± 0.5c	39.8 ± 4.9d	20.7 ± 0.7d	5.5 ± 0.3d
ANOVA (F values)										
N		4.55	0.38	31.1***	11.3**	0.57	1.59	44.0***	29.7***	151***
W		7.14**	20.1***	14.1**	14.9**	4	6.80*	47.4***	26.0***	52.0***
N*W		0.17	1.37	3.06	0.275	0.57	0.17	4.73*	0.34	5.42*

Values are mean ± SD. Duncan’s multiple-range test was used to test differences among treatments at the significance level of *P* < 0.05. The data in the table are the mean ± standard deviation of the observed values

Different lowercase letters within the same column denote significant differences among treatments. N1:110 kg ha^− 1^ nitrogen application, N2: 220 kg ha^− 1^ nitrogen application, W1: 45–55% FC, W2: 35–65% FC, and W3: 25–75% FC

*N*: Nitrogen, *W*: Irrigation limits, W*N: The interaction between nitrogen and irrigation limits, *s*: Seedling stage, *f*: Flowering period

*, **, and *** represent significance levels of *P* < 0.05, *P* < 0.01, and *P* < 0.001, respectively

Under the N1 condition, the W1 treatment significantly promoted okra growth compared to wider irrigation ranges (Table 2). At the seedling stage, plant height, stem diameter, and the number of expanded leaves under W1 were 4.5%, 10.5%, and 16.7% greater than under W2, and 8.3%, 21.9%, and 22.2% greater than under W3, respectively. This trend continued at the flowering stage. Furthermore, the dry weights of leaves, stems, and roots under W1 were significantly higher than those under W2 (by 12.8%, 7.1%, and 8.7%) and W3 (by 23.0%, 24.5%, and 34.8%). Under the N2 condition, the W1 treatment similarly enhanced plant growth (Table 2). The dry matter accumulation was particularly responsive; the weights of leaves, stems, and roots under W1 were 45.2%, 24.7%, and 26.7% higher, respectively, than under W3. Overall, although the expansion of irrigation upper and lower limits inhibited okra growth under both N conditions, plants under the N1 treatment consistently outperformed those under the N2 treatment. This advantage was particularly significant under the W3: compared to N2, the N1 treatment significantly increased plant height, leaf development, and biomass accumulation.

Analysis of N effects revealed that the N1 treatment generally outperformed N2. This advantage was most pronounced under the W1 condition, where N1 increased the dry weights of leaves, stems, and roots by 12.5%, 14.6%, and 34.8%, respectively, over N2.

### Yield and nitrogen partial factor productivity (NPFP)

**Fig. 3:**
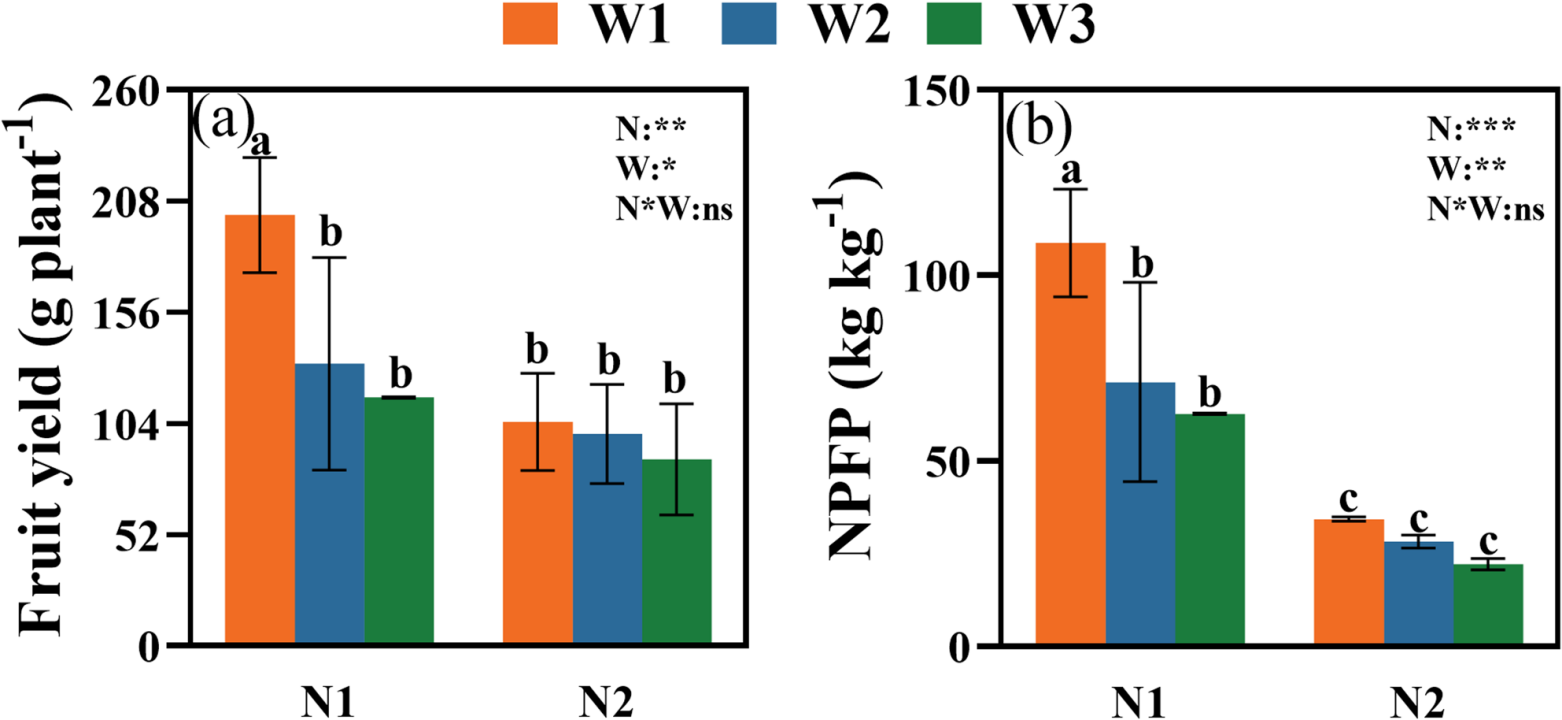
Effects of W and N application treatments on the fruit yield (**a**) and NPFP (**b**) of okra. Values are mean ± SD. Duncan’s multiple-range test was used to test differences among treatments at the significance level of *P* < 0.05. Different lowercase letters above the columns denote significant differences among treatments. N1:110 kg ha^− 1^ nitrogen application, N2: 220 kg ha^− 1^ nitrogen application, W1: 45–55% FC, W2: 35–65% FC, and W3: 25–75% FC. N: nitrogen, W: irrigation limits, W*N: the interaction between nitrogen and irrigation limits. *, **, and *** represent significance levels of *P* < 0.05, *P* < 0.01, and *P* < 0.001, respectively

Both irrigation and nitrogen significantly influenced fruit yield and NPFP, with a significant W×N interaction (Fig. 3).

The W1 treatment consistently yielded the highest values. Under N1, W1 increased fruit yield by 34.5% and 42.3% compared to W2 and W3, respectively (Fig. 3a). Similarly, under N2, W1 yield was 16.8% higher than W3.

The moderate nitrogen rate (N1) was more efficient than the excessive rate (N2). Under the optimal W1 condition, the N1 treatment increased yield by 48.0% and NPFP by 68.4% over N2 (Fig. 3a, b). This pattern of N1 superiority in enhancing both yield and NPFP was consistent across all irrigation treatments.

### Okra fruit quality

The quality of okra fruits was significantly affected by the main effects of W and N, and their interaction for most parameters (Table 3).

**Table 3:** Effects of W and N application treatments on fruit quality of Okra

Treatments		SS (mg g^− 1^)	SP (mg g^− 1^)	TSS (%)	TA (%)	F (mg g^− 1^)
N1	W1	4.08 ± 0.2a	3.85 ± 0.6a	9.9 ± 0.0a	0.07 ± 0.0b	13.4 ± 0.7b
	W2	3.59 ± 0.3bc	2.96 ± 0.2b	9.9 ± 0.0a	0.08 ± 0.0b	12.2 ± 0.4b
	W3	3.07 ± 0.1d	2.74 ± 0.2b	7.7 ± 1.6b	0.10 ± 0.0ab	15.1 ± 0.5a
N2	W1	3.82 ± 0.2ab	3.10 ± 0.5b	9.9 ± 0.0a	0.09 ± 0.0ab	12.9 ± 0.3b
	W2	3.32 ± 0.3 cd	3.06 ± 0.2b	6.6 ± 0.0b	0.09 ± 0.0ab	12.3 ± 0.8b
	W3	3.61 ± 0.1bc	3.01 ± 0.1b	6.6 ± 0.0b	0.12 ± 0.0a	14.6 ± 0.4a
ANOVA (F values)						
N		0.01	0.49	16.0**	3.34	1.02
W		10.8**	4.11*	19.0***	3.81	25.2***
N*W		5.56*	3.07	7.00*	0.05	0.39

Values are mean ± SD. Duncan’s multiple-range test was used to test differences among treatments at the significance level of *P* < 0.05. Different lowercase letters within the same column denote significant differences among treatments. N1:110 kg ha^− 1^ nitrogen application, N2: 220 kg ha^− 1^ nitrogen application, W1: 45–55% FC, W2: 35–65% FC, and W3: 25–75% FC

*N*: Nitrogen, *W*: Irrigation limits, W*N: The interaction between nitrogen and irrigation limits, *SS*: Soluble sugar, *SP*: Soluble protein, *TSS*: Total soluble solids, *TA*: Titratable acid, *F*: Flavonoid

*, **, and *** represent significance levels of *P* < 0.05, *P* < 0.01, and *P* < 0.001, respectively

Under N1, the W1 treatment significantly improved key quality indicators (Table 3). Compared to W3, W1 enhanced soluble sugars, soluble proteins, and total soluble solids by 8.3%, 21.9%, and 22.2%, respectively, while reducing titratable acid content by 42.9%.

The N1 treatment generally resulted in higher quality fruits than N2, particularly under the W1 condition. Here, N1 increased soluble sugar and soluble protein content by 6.4% and 19.5%, respectively, while reducing titratable acid by 28.6% compared to N2.

### Rhizosphere soil nutrient content

Soil nutrient content was significantly influenced by the main effects of W and N for most parameters, with several showing a significant W×N interaction (Table 4).

**Table 4:** Effects of W and N application treatments on the nutrient content of Okra cultivated soil

Treatments		TN (g kg^− 1^)	TP (%)	TK (%)	AP (mg kg^− 1^)	AK (mg kg^− 1^)	OM (g kg^− 1^)	AN (mg kg^− 1^)	pH	EC (µs cm^− 1^)
N1	W1	1.15 ± 0.0d	0.117 ± 0.0a	1.91 ± 0.0a	81.8 ± 0.7a	300 ± 20.9a	20.3 ± 0.6ab	8.09 ± 0.1bc	5.26 ± 0.2a	488 ± 12.1a
	W2	1.16 ± 0.0 cd	0.115 ± 0.0a	1.91 ± 0.0a	81.8 ± 1.3a	278 ± 13.5ab	20.4 ± 0.4a	7.24 ± 0.6c	4.91 ± 0.1b	475 ± 9.1ab
	W3	1.18 ± 0.0c	0.115 ± 0.0a	1.87 ± 0.0a	74.0 ± 1.9b	263 ± 6.7b	19.9 ± 0.2abc	7.25 ± 0.1c	4.93 ± 0.1b	453 ± 3.7b
N2	W1	1.23 ± 0.0b	0.116 ± 0.0a	1.89 ± 0.0a	68.0 ± 0.7c	250 ± 17.5b	19.3 ± 0.2 cd	9.25 ± 0.5a	4.68 ± 0.2bc	391 ± 8.4c
	W2	1.27 ± 0.0a	0.116 ± 0.0a	1.88 ± 0.0a	66.3 ± 0.9c	243 ± 8.7bc	19.4 ± 0.1bcd	9.03 ± 0.6ab	4.50 ± 0.1c	332 ± 21.9d
	W3	1.26 ± 0.0a	0.114 ± 0.0a	1.88 ± 0.0a	63.2 ± 0.9d	209 ± 13.7c	18.7 ± 0.6d	7.44 ± 0.6c	4.62 ± 0.0bc	357 ± 19.6d
ANOVA (F values)										
N		198***	0.35	1.36	393***	26.3***	21.5**	14.7**	33.1***	174***
W		10.5**	1.90	1.69	33.8***	6.23*	2.44	8.01**	4.61*	7.52*
N*W		1.77	0.79	1.67	4.23*	0.41	0.14	2.90	1.09	3.26

Values are mean ± SD. Duncan’s multiple-range test was used to test differences among treatments at the significance level of *P* < 0.05

Different lowercase letters within the same column denote significant differences among treatments

N1:110 kg ha^− 1^ nitrogen application, N2: 220 kg ha^− 1^ nitrogen application, W1: 45–55% FC, W2: 35–65% FC, and W3: 25–75% FC

*N*: Nitrogen, *W*: Irrigation limits, W*N: The interaction between nitrogen and irrigation limits, *TN*: Total nitrogen, *TP*: Total phosphorus, *TK*: Total potassium, *AP*: Available phosphorus, *AK*: Available potassium, *OM*: Organic matter, *AN*: Ammonium nitrogen, *pH*: Power of Hydrogen, *EC*: Electrical conductivity

*, **, and *** represent significance levels of *P* < 0.05, *P* < 0.01, and *P* < 0.001, respectively

The W1 treatment helped maintain a more favorable soil chemical environment. Under N1, W1 led to higher levels of available phosphorus (AP), available potassium (AK), and ammonium nitrogen (AN) compared to W3, with increases of 9.5%, 12.3%, and 10.4%, respectively (Table 4). Soil pH and EC were also higher under W1 than under W3 by 6.3% and 7.2%.

Notably, the N1 treatment was associated with better soil conditions than N2. Under W1, N1 increased soil pH and EC by 11.0% and 19.9%, respectively, over N2, while also leading to higher levels of organic matter (OM) and available nutrients.

### Soil enzyme activity

**Fig. 4:**
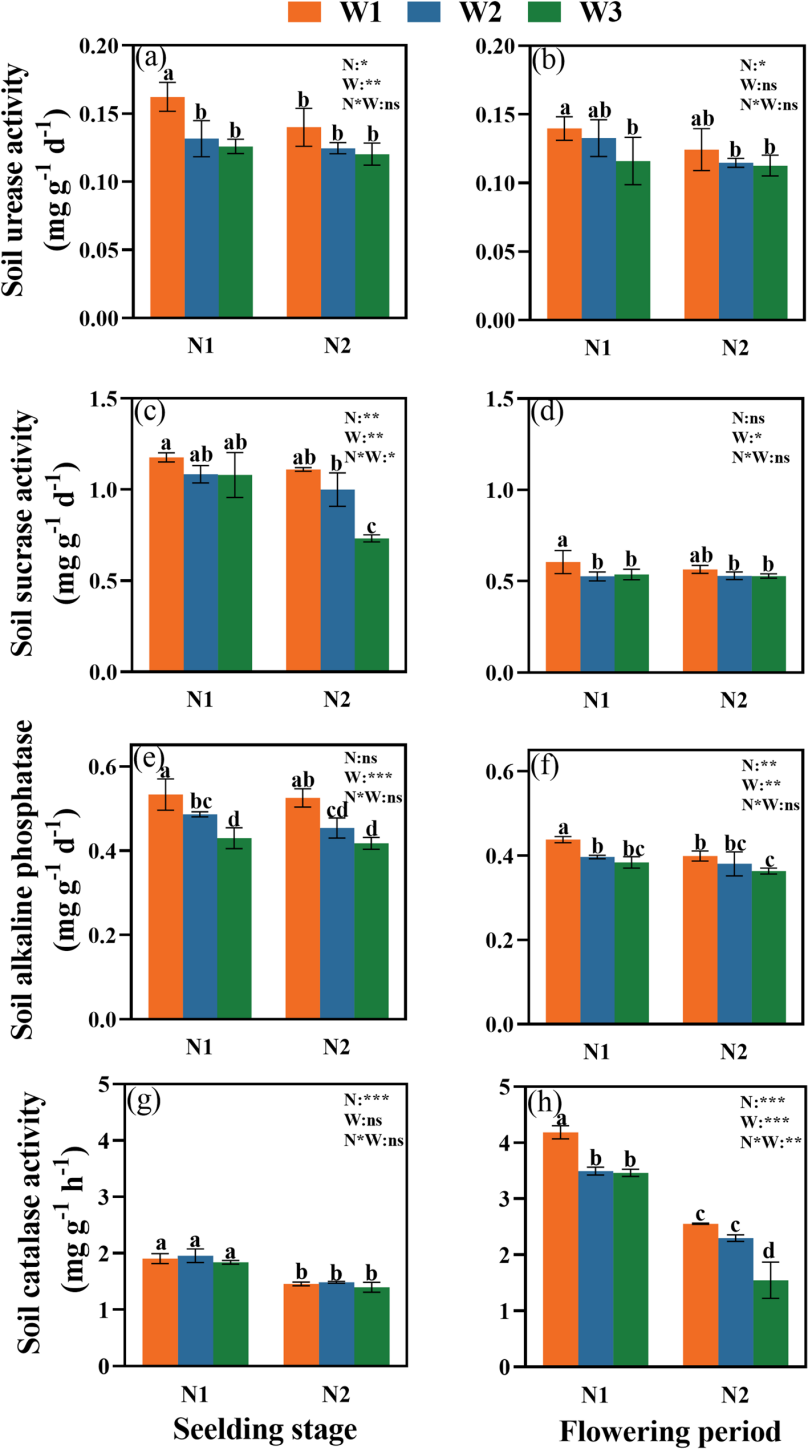
Effects of W and N application treatments on the soil urease activity, sucrase activity, alkaline phosphatase activity, and catalase activity. Values are mean ± SD. Duncan’s multiple-range test was used to test differences among treatments at the significance level of *P* < 0.05. Different lowercase letters above the columns denote significant differences among treatments. N1:110 kg ha^− 1^ nitrogen application, N2: 220 kg ha^− 1^ nitrogen application, W1: 45–55% FC, W2: 35–65% FC, and W3: 25–75% FC. N: nitrogen, W: irrigation limits, W*N: the interaction between nitrogen and irrigation limits. *, **, and *** represent significance levels of *P* < 0.05, *P* < 0.01, and *P* < 0.001, respectively

The activities of soil enzymes were significantly affected by W, N, and their interaction (W×N) at both growth stages (Fig. 4).

The W1 treatment consistently promoted greater soil enzyme activities. Under N1 at the flowering stage, the activities of urease, sucrase, alkaline phosphatase, and catalase under W1 were 17.1%, 11.2%, 12.3%, and 17.3% higher, respectively, than under W3 (Fig. 4).

The N1 treatment also positively influenced enzyme activity. Under the W1 condition at the flowering stage, N1 increased urease, sucrase, alkaline phosphatase, and catalase activities by 11.4%, 6.6%, 8.9%, and 39.0%, respectively, compared to N2.

### Correlation analysis

**Fig. 5:**
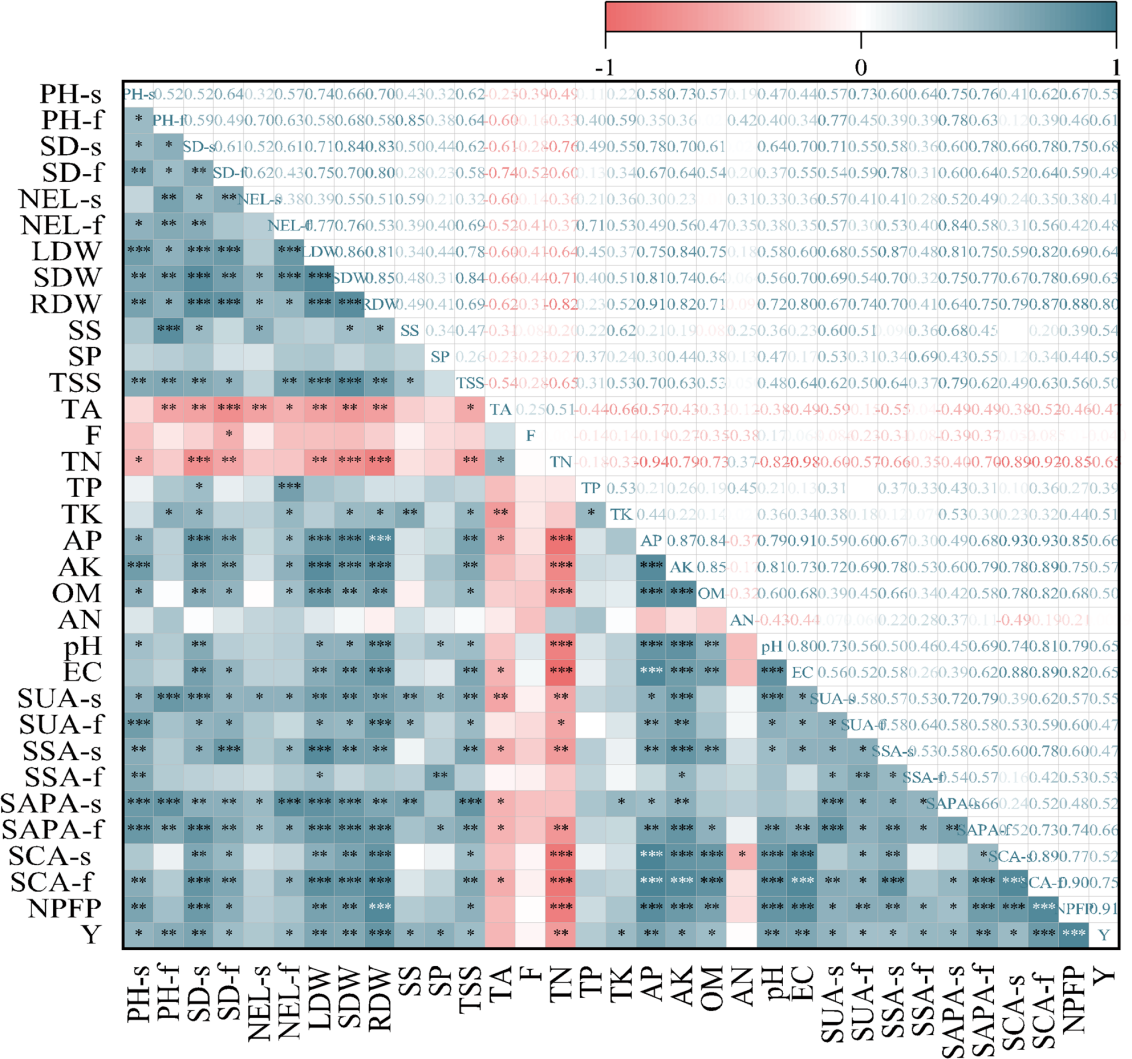
Correlation analysis between soil and plant indices. s, seedling stage; f, flowering period; PH, plant height; SD, stem diameter; NEL, number of expanded leaves; LDW, leaf dry weight; SDW, stem dry weight; RDW, root dry weight; SS, soluble sugar; SP, soluble protein; TSS, total soluble solids; TA, titratable acid; F, flavonoid; TN, total nitrogen; TP, total phosphorus; TK, total potassium; AP, available phosphorus; AK, available potassium; OM, organic matter; AN, ammonium nitrogen; pH, power of Hydrogen; EC, electrical conductivity; SUA, soil urease activity; SSA, soil sucrase activity; SAPA, soil alkaline phosphatase activity; SCA, soil catalase activity; NPFP, nitrogen fertilizer partial productivity; Y, fruit yield. *, ** and *** indicate significant differences at *P* < 0.05, *P* < 0.01 and *P* < 0.001 levels, respectively

Correlation analysis revealed key relationships between measured parameters (Fig. 5). Okra yield was significantly positively correlated with plant growth parameters and fruit quality components such as soluble sugars and soluble proteins. Yield was negatively correlated with soil total nitrogen (*r* = -0.651**). Furthermore, yield showed significant positive correlations with other soil physicochemical properties (e.g., AK, OM, pH, EC) and with all measured soil enzyme activities. The soil enzymes themselves were positively correlated with soil nutrients like available potassium, organic matter, and pH.

### Partial least squares path model (PLS-PM)

**Fig. 6:**
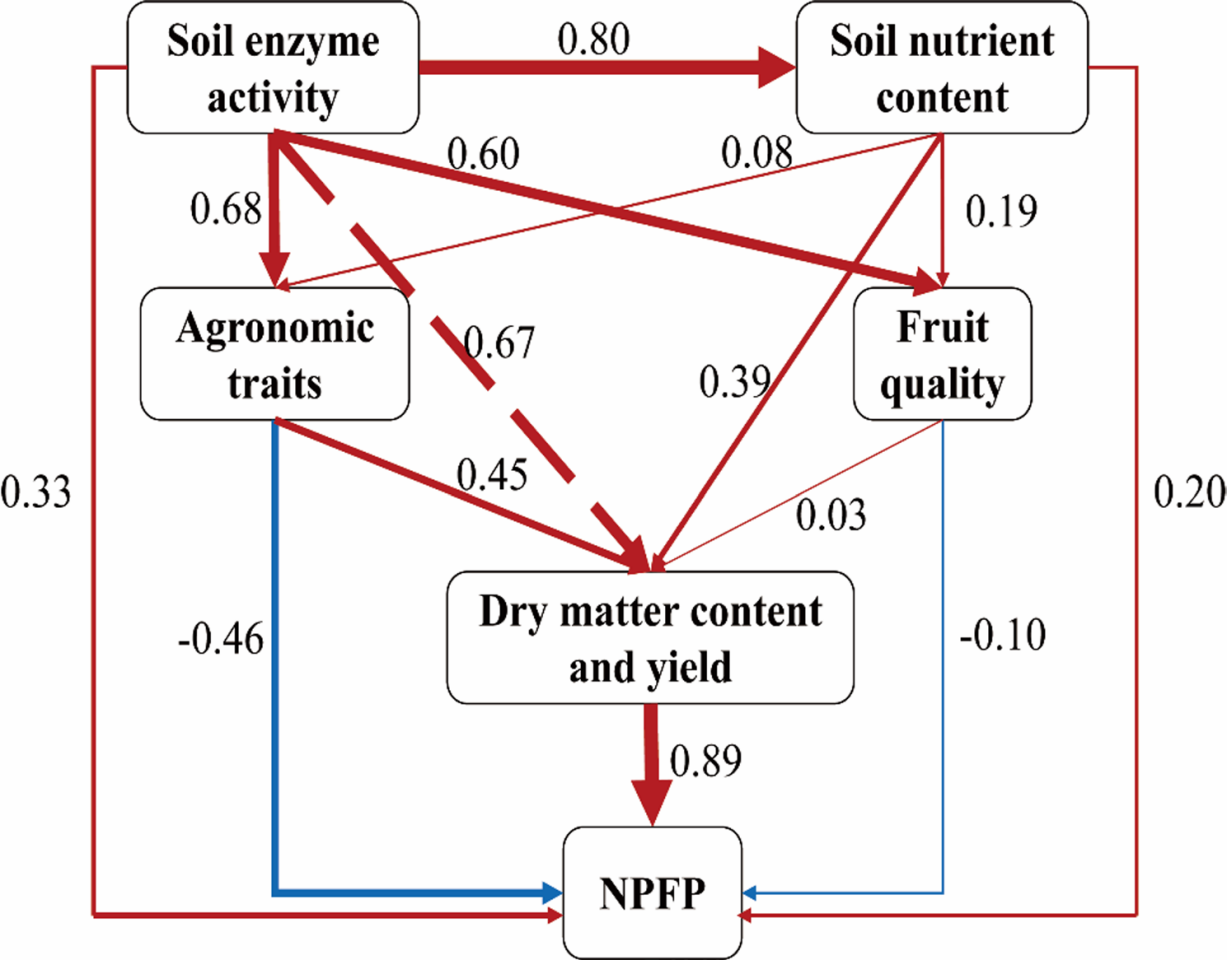
The partial least squares path modeling (PLS-PM) was employed to investigate the effects of varying nitrogen application rates and soil moisture on the yield of okra and the NPFP. The standardized path coefficients are indicated next to the arrows, with the width of the arrows being proportional to the strength of the association. Red arrows represent positive relationships, while blue arrows denote negative relationships. Solid arrows signify significant (*P* < 0.05) relationships, whereas dashed arrows indicate non-significant associations. The quality of the PLS-PM model is assessed by examining the goodness-of-fit index (GOF), which for this model is 0.71. The data utilized in the model pertain to the flowering stage

The PLS-PM analysis integrated the complex relationships among the studied variables (Fig. 6). The model had a good fit (Goodness-of-Fit = 0.71). The path coefficients revealed that soil enzyme activity exerted a strong positive direct effect on soil nutrient content (path coefficient = 0.80), agronomic traits (0.68), and fruit quality (0.60). Soil nutrient content, in turn, positively influenced agronomic traits, fruit quality, and ultimately yield and NPFP. Agronomic traits and fruit quality had a strong positive direct effect on yield and dry matter content, which then significantly determined NPFP (path coefficient = 0.89). This model illustrates that the positive effects of the W1 and N1 treatments on yield are mediated through enhanced soil biological activity (enzymes), improved soil nutrient availability, and superior plant growth and fruit quality.

## Discussion

### Synergistic enhancement of okra growth and nitrogen use efficiency by narrow irrigation range and moderate nitrogen

Optimal water and nitrogen (N) management is paramount for maximizing crop productivity [28, 60]. Our results demonstrate that maintaining a narrow irrigation limit range (W1) significantly enhances okra growth and yield compared to wider ranges (W2, W3) (Table 2; Fig. 3). Crucially, we found that a high irrigation upper limit could not compensate for the adverse effects of a low lower limit (Table 2). The growth suppression observed during low-moisture periods in the W2 and W3 treatments was not reversed by subsequent re-watering. This indicates that maintaining a narrow irrigation range, which ensures moisture stability, is the key driver for sustained okra growth. This finding aligns with studies on maize and tomato, where stable moisture regimes (often achieved through precise irrigation) consistently outperformed fluctuating ones in promoting root development and nutrient uptake [25, 26].

The benefits of the W1 treatment were maximized under moderate N application (N1). Under W1 conditions, N1 resulted in significantly better growth performance than excessive N application (N2) (Table 2), highlighting the importance of a balanced N supply for optimizing the benefits of a narrow irrigation range [58]. The synergistic N1W1 combination achieved the highest biomass, nitrogen partial factor productivity (NPFP), and yield among all treatments (Table 2; Fig. 3). Furthermore, under the stressful wide irrigation range (W3), the N1 treatment still outperformed N2 by significantly alleviating the adverse effects on growth and yield, demonstrating its superior efficacy across different moisture regimes. This underscores the critical role of optimal N supply. Studies on wheat have demonstrated that an adequate N level is pivotal under combined altered water regimes and different N treatments, whereas both deficiency and excess can be detrimental [24]. Our results confirm that balanced N application is essential to fully leverage the benefits of irrigation.

### Optimization of okra fruit quality through regulated water and nitrogen supply

Soil moisture critically influences fruit quality by modulating nutrient uptake and metabolic activity. Our study found that the W1 treatment significantly improved the contents of soluble sugars, proteins, and solids compared to W3 (Table 3). This supports our earlier conclusion that a high upper limit could not compensate for the adverse effects of a low lower limit. The wide fluctuations in soil moisture under W3 likely disrupted the steady supply of water and nutrients, impairing the consistent synthesis and accumulation of primary metabolites [20, 43].

Importantly, N1 alleviated the negative impact of the wide irrigation range (W3) on fruit quality. Under W3 conditions, the N1 treatment maintained higher levels of soluble sugars and proteins than N2 (Table 3). This suggests that balanced nitrogen nutrition helps sustain key metabolic pathways even under suboptimal moisture regimes [1].

A notable finding was the higher flavonoid content under W3. We propose that wide irrigation range induces premature fruit senescence. This accelerated aging process may trigger the accumulation of antioxidant compounds, including flavonoids, as a protective response to mitigate oxidative stress [53]. Similar stress-induced accumulation of secondary metabolites has been reported in other crops like tomatoes [45]. These findings collectively suggest that irrigation management strategies should be goal-oriented: a narrow irrigation range is optimal for maximizing overall fruit quality, whereas controlled moisture fluctuation can be a potential strategy to enrich specific functional compounds, such as flavonoids, through stress-induced metabolic reprogramming.

### Preservation of soil fertility and mitigation of acidification

Maintaining optimal soil moisture is essential for sustaining soil fertility, as it minimizes nutrient losses and preserves soil organic matter [51]. Our results showed that the W1 treatment significantly increased the levels of available phosphorus, rapidly available potassium, and ammonium N compared to W3 (Table 4). This reinforces the conclusion that the stability provided by W1 is irreplaceable; the higher irrigation upper limit in W3 failed to compensate for the nutrient retention deficits caused by its lower limit. Reduced leaching and enhanced retention of nutrients in the root zone under stable moisture likely contributed to this effect [13, 42]. Furthermore, W1 helped preserve higher soil organic matter levels, likely by minimizing the rapid mineralization pulses associated with frequent dry-wet cycles [32, 48].

N management also played a critical role in soil health. The excessive N2 rate, while increasing total N, led to significant soil acidification (Table 4). This is likely due to the nitrification processes associated with ammonium-based fertilizers [22]. Such acidification under N2 may subsequently decrease the availability of phosphorus and potassium and suppress microbial activity [9, 22]. In contrast, the moderate N1 rate maintained a more balanced pH and higher organic matter content, thereby supporting improved nutrient retention and availability. These findings highlight the importance of optimizing N application rates to maintain soil fertility, particularly under narrow irrigation range conditions.

### Narrow irrigation limit range promote soil enzyme activity

Soil enzyme activities are critical for nutrient cycling and microbial function, and they are strongly influenced by soil moisture. Our results showed that urease, sucrase, alkaline phosphatase, and catalase activities were significantly higher under the W1 condition compared to W3 (Fig. 4). This finding provides a mechanistic explanation for the earlier observation that a high irrigation upper limit could not compensate for a low lower limit. The stable and favorable moisture environment under W1 supports microbial growth and enzyme synthesis, whereas the fluctuating conditions in W3 disrupt these communities and biochemical processes [16, 18]. Importantly, N1 effectively mitigated the suppression of enzyme activities under the stressful W3 condition. The N1 treatment likely enhanced enzyme activities by improving microbial N availability. In contrast, the N2 treatment suppressed enzymatic functions, potentially due to the increased soil acidity observed (Table 4) or other imbalanced nutrient conditions [57].

### Narrow irrigation limit range enhances enzyme-mediated nutrient cycling

Soil enzymes mediate critical nutrient transformations, directly influence N, phosphorus, and carbon cycling in agroecosystems. Our findings show that under the W1 treatment significantly increased the activities of urease, sucrase, and alkaline phosphatase compared to W3 (Fig. 4). This suggests that maintaining a narrow irrigation range promotes enzyme-mediated nutrient cycling by sustaining microbial stability and optimizing soil chemical conditions.

Specifically, urease activity-a key enzyme in N cycling-was negatively correlated with soil ammonium N content (Fig. 5). This implies that stable moisture reduces the dependency on rapid N mineralization, thereby minimizing N losses and enhancing N availability [12, 47]. Similarly, sucrase activity, which facilitates carbon cycling by hydrolyzing sucrose, was positively correlated with soil organic matter levels. This reflects its vital role in generating carbon sources for microbial metabolism [54]. Furthermore, alkaline phosphatase activity, responsible for releasing plant-available phosphorus from organic compounds, was positively correlated with available phosphorus content. The stable moisture under W1 likely maintained optimal pH conditions, thereby promoting phosphorus solubilization and uptake [14]. Collectively, these relationships underscore the pivotal role of a narrow irrigation range in enhancing enzyme-mediated nutrient cycling and improving overall soil fertility.

### Limitations of the study and future perspectives

This study demonstrates that coupling a narrow irrigation range with moderate nitrogen application (N1W1) synergistically enhances okra growth, yield, fruit quality, and soil health by stabilizing the rhizosphere environment and optimizing nutrient cycling. While providing valuable insights, certain limitations of this work must be acknowledged. First, this was a pot experiment conducted under a rain-proof canopy in a single growing season. Although this setup allowed for precise irrigation control, the findings require validation under field conditions across multiple years and locations to account for broader environmental variability and larger-scale soil-plant-atmosphere interactions. Second, our assessment of soil health focused on chemical properties and enzyme activities. Future research should incorporate analyses of soil microbial community structure (e.g., via high-throughput sequencing) to gain a deeper, mechanistic understanding of how narrow irrigation range and appropriate N fertilizer application shape the microbiome that drives these processes. Finally, a comprehensive economic analysis is necessary to quantify the balance between savings in water and fertilizer inputs versus potential yield differences; changes in labor costs must also be incorporated. This integrated assessment of the overall economic benefit is pivotal for optimizing and promoting this management strategy.

## Conclusion

This study demonstrates that integrating a narrow irrigation range with moderate nitrogen (N) application constitutes the optimal strategy for enhancing the sustainability and productivity of okra cultivation. The principal findings are as follows:

(i) A high irrigation upper limit could not compensate for the detrimental effects of a low lower limit. A narrow range (W1: 45–55% FC) is crucial for maximizing plant growth and yield.
(ii) Moderate N application (N1: 110 kg ha⁻¹) is optimal, as it both enhances the benefits of W1 and helps alleviate the adverse effects of wide irrigation ranges.
(iii) The synergistic N1W1 combination simultaneously increased yield, improves key fruit quality parameters, enhances soil enzyme activities, and mitigated soil acidification.

Based on these results, we recommend adopting the integrated N1W1 management strategy. A primary limitation of this study is its controlled pot-based setting; future research should validate these findings under field conditions and further investigate the underlying soil microbial community dynamics.

## Data Availability

All data supporting the findings of this study are available within the paper and within its Supplementary Materials published online.

## References

[1] Abdelghany AE, Dou Z, Alashram MG, Eltohamy KM, Elrys AS, Liu X, Wu Y, Cheng M, Fan J, Zhang F. The joint application of Biochar and nitrogen enhances fruit yield, quality and water-nitrogen productivity of water-stressed greenhouse tomato under drip fertigation. Agric Water Manage. 2023;290:108605. 10.1016/j.agwat.2023.108605.

[2] Abid M, Tian Z, Ata-Ul-Karim ST, Cui Y, Liu Y, Zahoor R, Jiang D, Dai T. Nitrogen nutrition improves the potential of wheat (Triticum aestivum L.) to alleviate the effects of drought stress during vegetative growth periods. Front Plant Sci. 2016;7. 10.3389/fpls.2016.00981. 10.3389/fpls.2016.00981 PMC4927619 27446197

[3] Agami RA, Alamri SAM, Abd El-Mageed TA, Abousekken MSM, Hashem M. Role of exogenous nitrogen supply in alleviating the deficit irrigation stress in wheat plants. Agric Water Manage. 2018;210:261–70. 10.1016/j.agwat.2018.08.034.

[4] Aliku O, Oshunsanya SO. Modelling irrigation water requirements at physiological growth stages of Okra life cycle using CROPWAT model for derived Savannah and humid forest zones of Nigeria. Agric Trop Subtrop. 2016;49:20–9. 10.1515/ats-2016-0003.

[5] Arivalagan M, Karunakaran G, Roy TK, Dinsha M, Sindhu BC, Shilpashree VM, Satisha GC, Shivashankara KS. Biochemical and nutritional characterization of Dragon fruit (hylocereus species). Food Chem. 2021;353:129426. 10.1016/j.foodchem.2021.129426. 33774520 10.1016/j.foodchem.2021.129426

[6] Chadha A, Florentine SK, Chauhan BS, Long B, Jayasundera M. Influence of soil moisture regimes on growth, photosynthetic capacity, leaf biochemistry and reproductive capabilities of the invasive agronomic weed; lactuca serriola. PLoS ONE. 2019;14:e0218191. 10.1371/journal.pone.0218191. 31251746 10.1371/journal.pone.0218191 PMC6599151

[7] Chaturvedi AK, Surendran U, Gopinath G, Chandran KM, Nk A, Ct MF. Elucidation of stage specific physiological sensitivity of Okra to drought stress through leaf gas exchange, spectral indices, growth and yield parameters. Agric Water Manage. 2019;222:92–104. 10.1016/j.agwat.2019.05.041.

[8] Chen J, Song D, Luan H, Liu D, Wang X, Sun J, Zhou W, Liang G. Living and dead microorganisms in mediating soil carbon stocks under long-term fertilization in a rice-wheat rotation. Front Microbiol. 2022;13:854216. 10.3389/fmicb.2022.854216. 35756033 10.3389/fmicb.2022.854216 PMC9230992

[9] Chen L, Li KK, Shi WJ, Wang XL, Wang ET, Liu JF, Sui XH, Mi GH, Tian CF, Chen WX. Negative impacts of excessive nitrogen fertilization on the abundance and diversity of diazotrophs in black soil under maize monocropping. Geoderma. 2021;393:114999. 10.1016/j.geoderma.2021.114999.

[10] Chen L, Yang X, Huang X, Zhang X, Bai D, Ke Y. Effects of different nitrogen levels on the growth, yield and quality of Okra. Guangdong Agricultural Sci. 2016;43:77–81. 10.16768/j.issn.1004-874X.2016.05.015.

[11] Comas LH, Trout TJ, DeJonge KC, Zhang H, Gleason SM. Water productivity under strategic growth stage-based deficit irrigation in maize. Agric Water Manage. 2019;212:433–40. 10.1016/j.agwat.2018.07.015.

[12] Davies B, Coulter JA, Pagliari PH. Soil enzyme activity behavior after Urea nitrogen application. Plants. 2022;11:2247. 10.3390/plants11172247. 36079628 10.3390/plants11172247 PMC9460541

[13] Denef K, Six J, Bossuyt H, Frey SD, Elliott ET, Merckx R, Paustian K. Influence of dry–wet cycles on the interrelationship between aggregate, particulate organic matter, and microbial community dynamics. Soil Biol Biochem. 2001;33:1599–611. 10.1016/S0038-0717(01)00076-1.

[14] Dick WA, Cheng L, Wang P. Soil acid and alkaline phosphatase activity as pH adjustment indicators. Soil Biol Biochem. 2000;32:1915–9. 10.1016/S0038-0717(00)00166-8.

[15] Ding Y, Xu J. Global vulnerability of agricultural commodities to climate risk: evidence from satellite data. Econ Anal Policy. 2023;80:669–87. 10.1016/j.eap.2023.09.013.

[16] Gao D, Bai E, Wasner D, Hagedorn F. Global prediction of soil microbial growth rates and carbon use efficiency based on the metabolic theory of ecology. Soil Biol Biochem. 2024;190:109315. 10.1016/j.soilbio.2024.109315.

[17] Gou W, Zheng P, Tian L, Gao M, Zhang L, Akram NA, Ashraf M. Exogenous application of Urea and a Urease inhibitor improves drought stress tolerance in maize (zea Mays L). J Plant Res. 2017;130:599–609. 10.1007/s10265-017-0933-5. 28324190 10.1007/s10265-017-0933-5

[18] Han D, Zhang D, Han, Dezhi, Ren H, Wang Z, Zhu Z, Sun H, Wang L, Qu Z, Lu W, Yuan M. Effects of salt stress on soil enzyme activities and rhizosphere microbial structure in salt-tolerant and -sensitive soybean. Sci Rep. 2023;13:17057. 10.1038/s41598-023-44266-5. 37816809 10.1038/s41598-023-44266-5 PMC10564926

[19] Han JM, Flexas J, Xiong DL, Galmés J, Zhang YL. Regulation of photosynthesis and water-use efficiency in Pima and upland cotton species subjected to drought and recovery. Photosynthetica. 2024;62:6–15. 10.32615/ps.2023.036. 39650630 10.32615/ps.2023.036 PMC11609770

[20] Han L, Nan G, He X, Wang J, Zhao J, Zhang X. Soil moisture and soil organic carbon coupled effects in Apple orchards on the loess plateau, China. Sci Rep. 2024;14:12281. 10.1038/s41598-024-63039-2. 38811638 10.1038/s41598-024-63039-2 PMC11136960

[21] Han X, Cheng Z, Meng H. Soil properties, nutrient dynamics, and soil enzyme activities associated with Garlic stalk decomposition under various conditions. PLoS ONE. 2012;7:e50868. 10.1371/journal.pone.0050868. 23226411 10.1371/journal.pone.0050868 PMC3511307

[22] Hu Z, Delgado-Baquerizo M, Fanin N, Chen X, Zhou Y, Du G, Hu F, Jiang L, Hu S, Liu M. Nutrient-induced acidification modulates soil biodiversity-function relationships. Nat Commun. 2024;15:2858. 10.1038/s41467-024-47323-3. 38570522 10.1038/s41467-024-47323-3 PMC10991381

[23] Kachurina OM, Zhang H, Raun WR, Krenzer EG. Simultaneous determination of soil aluminum, ammonium- and nitrate-nitrogen using 1 *M* potassium chloride extraction. Commun Soil Sci Plant Anal. 2000;31:893–903. 10.1080/00103620009370485.

[24] Kartseva T, Dobrikova A, Kocheva K, Alexandrov V, Georgiev G, Brestič M, Misheva S. Optimal nitrogen supply ameliorates the performance of wheat seedlings under osmotic stress in genotype-specific manner. Plants. 2021;10:493–511. 10.3390/plants10030493. 33807753 10.3390/plants10030493 PMC7999466

[25] Li G, Long H, Zhang R, Drohan PJ, Xu A, Niu L. Stable soil moisture alleviates water stress and improves morphogenesis of tomato seedlings. Horticulturae. 2023;9:391. 10.3390/horticulturae9030391.

[26] Li G, Long H, Zhang R, Xu A, Niu L. Photosynthetic traits, water use and the yield of maize are influenced by soil water stability. BMC Plant Biol. 2024;24. 10.1186/s12870-024-05942-4. 10.1186/s12870-024-05942-4 PMC11665097 39710671

[27] Li G, Zhu G, Liu J, Wang Z, Long H, Zhang R, Yu K. Effects of stable and fluctuating soil water on the agronomic and biological performance of root vegetables. Front Plant Sci. 2024b;15:1325078. 10.3389/fpls.2024.1325078. 38419780 10.3389/fpls.2024.1325078 PMC10899879

[28] Li N, Wang X-X, Xue Z, Li Q. Water and potassium utilization efficiency and yield and quality of cucumber (cucumis sativus L). Sci Hortic. 2024;330:113025. 10.1016/j.scienta.2024.113025.

[29] Li T, Liu Y, Ye J, Wang S, Yin L, Deng X, Shan L. Response and mechanism of source-sink-flow caused by the compensation effect of crop rehydration after drought. J Soi Land Water Conserv. 2024;38:1–12. 10.13870/j.cnki.stbcxb.2024.02.002.

[30] Li Y, Fang F, Wei J, Wu X, Cui R, Li G, Zheng F, Tan D. Humic acid fertilizer improved soil properties and soil microbial diversity of continuous cropping peanut: a three-year experiment. Sci Rep. 2019;9:12014. 10.1038/s41598-019-48620-4. 31427666 10.1038/s41598-019-48620-4 PMC6700118

[31] Li Z, Huang J, Zhang Y, Wang Q, Zhou Y. Replacing common Urea with controlled-release fertilizer at the recommended rate increases the yield and nitrogen fertilizer productivity of sorghum. J Plant Nutr Fertil. 2024;30:49–62.

[32] Linn DM, Doran JW. Effect of water-filled pore space on carbon dioxide and nitrous oxide production in tilled and nontilled soils. Soil Sci Soc Am J. 1984;48:1267–72. 10.2136/sssaj1984.03615995004800060013x.

[33] Liu L, Gudmundsson L, Hauser M, Qin D, Li S, Seneviratne SI. Soil moisture dominates dryness stress on ecosystem production globally. Nat Commun. 2020;11:4892. 10.1038/s41467-020-18631-1. 32994398 10.1038/s41467-020-18631-1 PMC7524720

[34] Ndabankulu K, Egbewale SO, Tsvuura Z, Magadlela A. Soil microbes and associated extracellular enzymes largely impact nutrient bioavailability in acidic and nutrient poor grassland ecosystem soils. Sci Rep. 2022;12:12601. 10.1038/s41598-022-16949-y. 35871260 10.1038/s41598-022-16949-y PMC9308775

[35] Niu L, Wang Z, Zhu G, Yu K, Li G, Long H. Stable soil moisture improves the water use efficiency of maize by alleviating short-term soil water stress. Front Plant Sci. 2022;13:833041. 10.3389/fpls.2022.833041. 35519805 10.3389/fpls.2022.833041 PMC9062231

[36] Prajapati HA, Yadav K, Hanamasagar Y, Kumar MB, Khan T, Belagalla N, Thomas V, Jabeen A, Gomadhi G, Malathi G. Impact of climate change on global agriculture: challenges and adaptation. Int J Environ Clim Change. 2024;14:372–9. 10.9734/ijecc/2024/v14i44123.

[37] Qi M, Liu X, Li Y, Song H, Yin Z, Zhang F, He Q, Xu Z, Zhou G. Photosynthetic resistance and resilience under drought, flooding and rewatering in maize plants. Photosynth Res. 2021;148:1–15. 10.1007/s11120-021-00825-3. 33661466 10.1007/s11120-021-00825-3

[38] Ridhuan AUM, Rahman RA. Study of soil quality (moisture and pH) upon the growth of Okra plant using IoT technique . 2022;3:290–7. 10.30880/rpmme.2022.03.01.030.

[39] Saiya-Cork KR, Sinsabaugh RL, Zak DR. The effects of long term nitrogen deposition on extracellular enzyme activity in an Acer saccharum forest soil. Soil Biol Biochem. 2002;34:1309–15. 10.1016/S0038-0717(02)00074-3.

[40] Sanchez G, Trinchera L, Russolillo G. plspm: tools for partial least squares path modeling (PLS-PM). R Package Version 0.4 7. 2015. Available via GitHub. https://github.com/gastonstat/plspm.

[41] Sardans J, Peñuelas J. Drought decreases soil enzyme activity in a mediterranean Quercus ilex L. forest. Soil Biol Biochem. 2005;37:455–61. 10.1016/j.soilbio.2004.08.004.

[42] Schjønning P, Elmholt S, Munkholm LJ, Debosz K. Soil quality aspects of humid sandy loams as influenced by organic and conventional long-term management. Agric Ecosyst Environ. 2002;88:195–214. 10.1016/S0167-8809(01)00161-X.

[43] Seo H-J, Sawant SS, Lee B, Kim K, Song J, Choi ED. Mechanisms driving fruit cracking in ‘sinhwa’ Pears (pyrus pyrifolia nakai) and effect of foliar fertilizer application on fruit quality. Sci Hortic. 2024;332:113232. 10.1016/j.scienta.2024.113232.

[44] Serna-Escolano V, Giménez M, Serrano M, Valero D, García-Pastor M, Dobón-Suarez A, Gutiérrez-Pozo M, Giménez-Berenguer M, Zapata P. Fruit position on tree canopy affects fruit quality traits in ‘sanguinelli’ blood oranges. Horticulturae. 2024;10:949–62. 10.3390/horticulturae10090949.

[45] Sharma D, Shree B, Kumar S, Kumar V, Sharma, Shweta, Sharma S. Stress induced production of plant secondary metabolites in vegetables: functional approach for designing next generation super foods. Plant Physiol Biochem. 2022;192:252–72. 10.1016/j.plaphy.2022.09.034. 36279745 10.1016/j.plaphy.2022.09.034

[46] Shen W, Lin X, Gao N, Zhang H, Yin R, Shi W, Duan Z. Land use intensification affects soil microbial populations, functional diversity and related suppressiveness of cucumber fusarium wilt in china’s Yangtze river delta. Plant Soil. 2008;306:117–27. 10.1007/s11104-007-9472-5.

[47] Sigua GC, Novak JM, Watts DW, Myers WT, Ducey TF, Stone KC. Urease activity and nitrogen dynamics in highly weathered soils with designer biochars under corn cultivation. Biochar. 2020;2:343–56. 10.1007/s42773-020-00052-4.

[48] Six J, Bossuyt H, Degryze S, Denef K. A history of research on the link between (micro)aggregates, soil biota, and soil organic matter dynamics. Soil Tillage Res. 2004;79:7–31. 10.1016/j.still.2004.03.008.

[49] Sneha GR, Swarnalakshmi K, Sharma M, Reddy K, Bhoumik A, Suman A, Kannepalli A. Soil type influence nutrient availability, microbial metabolic diversity, eubacterial and Diazotroph abundance in Chickpea rhizosphere. World J Microbiol Biotechnol. 2021;37:167. 10.1007/s11274-021-03132-0. 34468874 10.1007/s11274-021-03132-0

[50] Soothar RK, Singha A, Soomro SA, Chachar A, Kalhoro F, Rahaman MA. Effect of different soil moisture regimes on plant growth and water use efficiency of sunflower: experimental study and modeling. Bull Natl Res Cent. 2021;45:121. 10.1186/s42269-021-00580-4.

[51] Szejgis J, Nielsen UN, Dijkstra FA, Carrillo Y. Prolonged drought moderates flood effects on soil nutrient pools across a rainfall gradient. Soil Biol Biochem. 2024;193:109404. 10.1016/j.soilbio.2024.109404.

[52] Udpuay S, Ullah H, Himanshu SK, Tisarum R, Praseartkul P, Cha-um S, Datta A. Effects of microbial biofertilizer on growth, physio-biochemical traits, fruit yield, and water productivity of Okra under drought stress. Biocatal Agric Biotechnol. 2024;58:103125. 10.1016/j.bcab.2024.103125.

[53] Wadood A, Hameed A, Akram S, Ghaffar M. Unraveling the impact of water deficit stress on nutritional quality and defense response of tomato genotypes. Front Plant Sci. 2024;15:1403895. 10.3389/fpls.2024.1403895. 38957600 10.3389/fpls.2024.1403895 PMC11217520

[54] Wang L, Luo N, Shi Q, Sheng M. Responses of soil labile organic carbon fractions and enzyme activities to long-term vegetation restorations in the karst ecosystems, Southwest China. Ecol Eng. 2023;194:107034. 10.1016/j.ecoleng.2023.107034.

[55] Wang X, Zhao F, Wu Y. Increased sensitivity of vegetation to soil moisture and its key mechanisms in the loess plateau. China Ecohydrology. 2024;17:e2602. 10.1002/eco.2602.

[56] Wu W, Dong X, Chen G, Lin Z, Chi W, Tang W, Yu J, Wang S, Jiang X, Liu X, Wu Y, Wang, Chunyuan, Cheng X, Zhang W, Xuan W, Terzaghi W, Ronald PC, Wang H, Wang, Chunming, Wan J. The elite haplotype OsGATA8-H coordinates nitrogen uptake and productive tiller formation in rice. Nat Genet. 2024;56:1516–26. 10.1038/s41588-024-01795-7. 38872029 10.1038/s41588-024-01795-7 PMC11250373

[57] Xu M, Fan L, Li A, Liu Q, Yu G, Wang S, Zhang B, Ye Q, Mo J, Zheng M. Plant and microbial carbon are important drivers of free-living nitrogen fixation in tropical forest soils: a new discovery of carbon‐driven nitrogen input. Geophys Res Lett. 2024;51:e2024GL111238. 10.1029/2024GL111238.

[58] Xu S, Huang Y, Zhang R, Niu L, Long H. Appropriate nitrogen application for alleviation of soil moisture-driven growth Inhibition of Okra (abelmoschus esculentus L. (moench)). Horticulturae. 2024;10:425. 10.3390/horticulturae10050425.

[59] Zhang J, Jin K, Luo Y, Du L, Tian R, Wang S, Shen Y, Zhang, Jiatao, Li N, Shao W, Xu Z. Responses of soil enzyme activity to long-term nitrogen enrichment and water addition in a typical steppe. Agronomy. 2023;13:1920. 10.3390/agronomy13071920.

[60] Zhou H, Niu X, Yan H, Zhao N, Zhang F, Wu L, Yin D, Kjelgren R. Interactive effects of water and fertilizer on Yield, soil water and nitrate dynamics of young Apple tree in semiarid region of Northwest China. Agronomy. 2019;9:360. 10.3390/agronomy9070360.

[61] Zhu P, Jia X, Zhao C, Shao M. Long-term soil moisture evolution and its driving factors across china’s agroecosystems. Agric Water Manage. 2022;269:107735. 10.1016/j.agwat.2022.107735.

